# A brief novel questionnaire to estimate premorbid functional state in acute stroke patients

**DOI:** 10.1007/s10072-026-09133-x

**Published:** 2026-06-01

**Authors:** Askiel Bruno, Giandor Saltz, Matthew L. Abbott, Mithilesh Siddu, Aisha Naushad, Vetriganesh Maduraiveeran, Justin Skariah, Kareena Udeshi, Saad Tanzeem, Bruno Quevedo-Tejada, Adarshini Raja, Kevin Dobbin, Fenwick T. Nichols

**Affiliations:** 1 Department of Neurology, Medical College of Georgia at Augusta University, 1120 15th Street, Augusta, GA 30912 Georgia; 2 Medical College of Georgia, Augusta University, Augusta , Georgia; 3 Department of Biostatistics, Data Science & Epidemiology, School of Public Health, Augusta University, Augusta, Georgia

**Keywords:** Activities of daily living, Disabilities, Functional state, Stroke

## Abstract

**Background:**

During acute stroke evaluation, knowing a patient’s premorbid functional state can guide clinical decision and research considerations. The often-utilized modified Rankin scale was not designed and is not well suited for this purpose. Thus, we developed and tested a brief questionnaire to assess a patient’s premorbid functional state.

**Methods:**

The novel questionnaire scores range from 0 to 5. Scores 0–2 represent increasing degrees of independent functioning, not at all difficult (0), somewhat difficult (1), and very difficult (2). Scores 3, 4, and 5 represent moderate to severe disabilities. Paired raters independently tested the novel questionnaire on two consecutive days in patients with acute cerebrovascular events. The kappa statistic evaluated reliability of the novel questionnaire and previously established questionnaires were used for concurrent validity testing.

**Results:**

In 76 patients, mean age 77 (SD 14) years and 51% women, the overall agreement between the paired raters was 79%. The standard kappa was 0.62 (95% CI 0.47–0.76), *p* < 0.001, and the weighted kappa was 0.89 (95% CI 0.82–0.96), *p* < 0.001. The novel questionnaire scores correlated well with the Groningen scale (*r* = 0.70 and *r* = 0.67 for the two raters, *p* < 0.001 for both), and with the Lawton scale (*r* = -0.77 and *r*= -0.82 for the two raters, *p* < 0.001 for both).

**Conclusions:**

This novel brief baseline function questionnaire for assessing a patient’s premorbid functional state has acceptable clinimetric properties and could be used to rapidly and reliably score the premorbid baseline function among acute stroke patients.

## Introduction

During the initial evaluation of patients with acute stroke, rapid determination of premorbid level of function is useful in guiding optimal clinical management and determining eligibility for clinical research. Lower levels of independence before a stroke, such as limitations in instrumental activities of daily living, are associated with less recovery of function and reduced likelihood of returning home [1]. The commonly used modified Rankin scale to estimate the premorbid baseline function in acute stroke patients is not well suited for this purpose [2]. For scoring mild disabilities, the mRS requires a comparison between a baseline and a subsequent state after a brain injury, and before a stroke only one state is available, the baseline state.

Ideally, during an acute stroke evaluation, a premorbid function assessment tool should be brief, reliable, accurate, and indicate global functioning. Although many instruments are currently available to assess basic [3] and instrumental [4] activities of daily living, none of them poses all these desired qualities.

Thus, we developed and tested a novel brief baseline function questionnaire for use during acute stroke. Our original questionnaire had suboptimal reliability for clinical use [5]. Here, we report the reliability and validity of our revised brief baseline function questionnaire.

## Methods

For content validity, two senior vascular neurologists (A.B and F.T.N) revised the original version of this brief baseline function questionnaire [5] to better quantify mild degrees of premorbid disabilities among patients hospitalized with acute ischemic or hemorrhagic stroke, or transient ischemic attack. The first question asks about the ability to live alone and is adopted from the simplified modified Rankin Scale questionnaire (smRSq) [6]. Because the mild mRS disability scores 0–2 are not suitable for baseline function assessment, we developed a novel closed-ended question prompting a three-level response in patients reporting independent functioning, not at all difficult (0), somewhat difficult (1), and very difficult (2), (Figure). Because the moderate-severe mRS disability scores 3–5 are suitable for baseline function assessment, we adopted the smRSq questions for this purpose. Thus, the novel questionnaire scores range from 0 to 5.

Eleven raters participated. Two raters blinded to each other’s results, separately interviewed each participant on two consecutive days. Different days over same day interviews were used to limit recall bias. The two raters for each participant were selected based on availability. Both interviews were completed within 3 days of hospital admission. In addition to the novel questionnaire, for concurrent validation, the first rater scored the basic and instrumental Groningen Activity Restriction Scale,^7^ and the second rater scored the Lawton Instrumental Activities of Daily Living scale [7]. Once scored, all scores were final. We did not compare the novel scale to the mRS because the mRS is not suitable for scoring mild degrees of premorbid disability (0–2).^2^

**Fig. 1:**
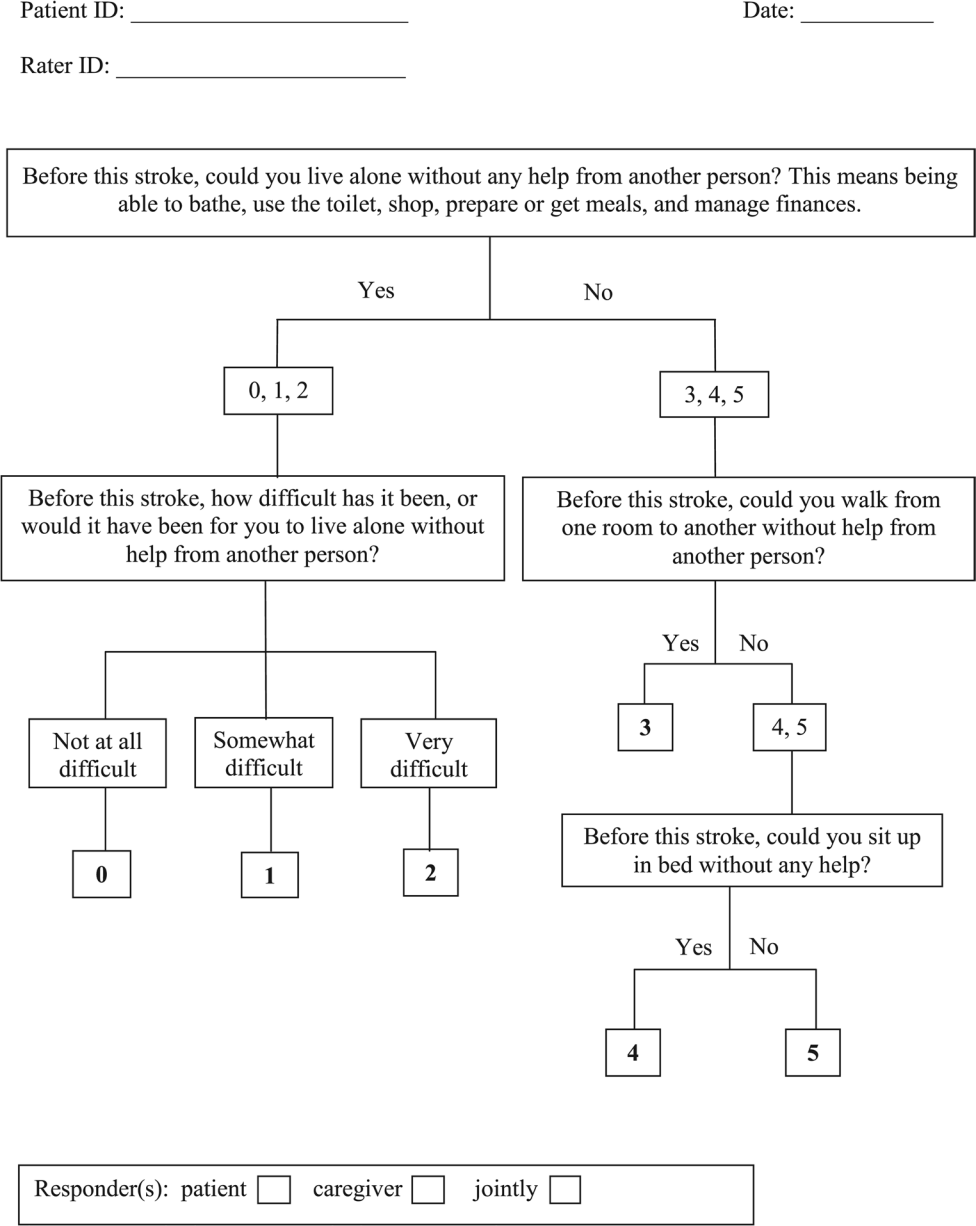
Brief baseline function questionnaire

Questioning started with the first (top) question in Fig. 1. Depending on the first answer, either the left or the right next in line algorithm question was asked. The second left algorithm question is the novel closed-ended question prompting a three-level response. The answer was circled as the final answer. The second right algorithm question asks about independent walking and a “yes” response ended this questionnaire and the score of 3 was circled. A “no” response to this second question lead to a third right algorithm question about ability to sit up in bed without assistance, and a “yes” response prompted a score of 4 while a “no” response prompted a score of 5, and the final answer was circled.

As an example, a participant who can independently bathe, use the toilet, shop, prepare or get meals, and manage finances despite some difficulties would answer “yes” to the first algorithm question. Then the answer to the second and final left algorithm question should be “somewhat difficult” indicating a score of 1 on this scale.

Because patients with acute stroke deficits may have been unable to participate and their clinical condition was at risk of worsening, we included caregivers as responders to the questionnaire when feasible. Every effort was made to interview the same individual(s) during each of the paired interviews. This protocol was approved by the Augusta University Institutional Review Board in accordance with the Declaration of Helsinki 2024, and all participants signed a valid informed consent agreeing to the two interviews.

After enrolling the initial 35 participants, we noticed a skewed distribution of scores towards 0 (not at all difficult). Therefore, at that time we modified this protocol to recruit only patients aged 80 years or older to facilitate a more balanced distribution of scores 0–2.

The kappa and weighted kappa statistics tested this questionnaire for reliability as the scores are ordinal [8]. We used standard inverse square distances for the weights. For concurrent validity, Spearman regression compared the novel questionnaire ordinal scores to the Groningen and the Lawton scales scores. Plots showed roughly linear relationships between the novel scores and the Groningen and Lawton scores (not shown).

## Results

We enrolled 80 patients and four could not be interviewed a second time, leaving 76 with full data for analysis. Their mean age was 77 (SD 14) years and 51% were women. Majority of the patients reported ability to live alone before their hospitalization, 63/76 (83%) for the first rater and 62/76 (82%) for the second rater.

The paired raters’ scores agreed for 60 of 76 participants (79%). The standard kappa showed substantial agreement of 0.62 (95% CI 0.47–0.76), *p* < 0.001. The weighted kappa showed excellent agreement of 0.89 (95% CI 0.82–0.96), *p* < 0.001, after accounting for the extent of disagreements. Table 1 shows the overall cross-tabulation of the scores by the paired raters.

**Table 1:** Cross-tabulation of the novel questionnaire scores by two paired raters

	Rater 2					
Rater 1	0	1	2	3	4	Total
0	**45**	2	0	0	0	47
1	5	**5**	1	2	0	13
2	0	1	**1**	1	0	3
3	0	1	1	**8**	1	11
4	0	0	0	1	**1**	2
Total	50	9	3	12	2	**76**

The agreements differed by responder type. When the patients alone were the responders, agreement was 89% (25/28) and the standard kappa was 0.75 (weighted kappa 0.69). When caregivers alone were the responders, agreement was 77% (17/22) and the standard kappa was 0.64 (weighted kappa 0.95). When the responses were provided jointly, agreement was 60% (6/10) and the standard kappa 0.33 (weighted kappa 0.71). For 14 participants the responders differed between the two interviews or their identity was missing.

Inter-rater agreements and reliability results also differed between the two study phases, the initial and the later phase after limiting enrollment to patients 80 years or older (Table 2). Agreements and reliability statistics were highest among the younger 35 participants in the first phase of this study.

**Table 2:** Participant characteristics and reliability between the initial and the later phase of study

Characteristic	Overall (*N* = 76)	Initial subgroup (*n* = 34)	Later ≥ 80 years subgroup (*n* = 42)
Mean age, yrs (SD)	77 (14)	66 (14)	85 (4)
Women (%)	39 (51)	17 (50)	23 (55)
Inter-rater % agreement	79 (60/76)	88 (30/34)	71 (30/42)
Standard kappa	0.62	0.71	0.55
Weighted kappa	0.89	0.92	0.85

In validity testing, there was moderate to high correlation between the novel questionnaire and the Groningen scale, r **=** 0.70 and *r* = 0.67 for the first and second rater, respectively, *p* < 0.001 for both. There was high correlation between the novel questionnaire and the Lawton scale, *r* = -0.76 and *r*= -0.77, for the first and second rater, respectively, *p* < 0.001 for both.

## Discussion

We tested a novel brief baseline function questionnaire designed to reliably score the premorbid global function in patients with acute stroke. Such information can help in determining optimal clinical management and establishing eligibility for clinical research.

The overall inter-rater agreement (79%) and the standard kappa reliability statistic (0.62) of the novel questionnaire are substantial with moderate to high concurrent validity. This questionnaire fills an important current gap in the acute assessment of stroke patients and could be used to rapidly and reliably assess global pre-morbid functional state. Although in this study we did not monitor the time to administer this questionnaire, the similar smRSq takes an average of 1.5 min to score.

One advantage of this novel questionnaire is the overlap with the familiar smRSq,^6^ both questionnaires having the same criteria for grading moderate-severe disability scores 3–5. However, we caution against comparing the novel questionnaire scores to the post-stroke smRSq scores as the questions for scoring 0–2 are different between these questionnaires.

The reliability of this novel questionnaire was considerably higher when patients alone were the responders (89%) than when caregivers alone were the responders (77%), or when the responses were provided jointly (60%). Possibly, due to the subjective nature of the answers scoring 0–2 (not at all difficult, somewhat difficult, very difficult), patients were more confident and accurate responders than the caregivers and were less likely to reconsider their answers during the second interview. In addition, the sample size diminishes to 22 for caregivers alone and to 10 for joint responders, making these results less reliable. Until additional data suggest otherwise, it seems best to rely on the patients’ responses to this questionnaire when feasible.

The moderate to high correlation between the novel questionnaire and established scales of basic and instrumental activities of daily living [7, 9] indicates that the novel questionnaire largely measures the intended item. As with the modified Rankin scale, the novel questionnaire indicates a person’s global functional state but is specifically suited for premorbid assessment.

One limitation in this study is skewness of the scores towards zero, limiting the evaluation of the entire novel 0–2 score range, and creating a prevalence effect that could have artificially lowered the kappa values [10]. This could be overcome in future studies by revising the relevant questions or including participants more likely to have difficulties living independently. A second potential limitation is that a recall bias could have amplified the reliability results. This limitation seems to apply more to the patients than to the caregivers as responders since the patients had nearly perfect agreement between the two interviews. This limitation could be reduced in future studies by delaying the second interview. A third potential limitation to reliability testing is using different responders between the two interviews. We minimized this limitation by making every effort to interview the same responders during the two interviews. In addition, we recommend a subsequent larger and preferably multicenter study with a broader distribution of scores to validate our current findings.

## Conclusion

The novel brief baseline function questionnaire we tested shows substantial reliability and high concurrent validity and fills an important current gap in the assessment of acute stroke patients. Valuable advantages over existing scales include simplicity, brevity, assessment of global function, and overlap with the familiar smRSq for scoring moderate to severe premorbid disabilities.

## Data Availability

The data from this study may be shared with qualified investigators upon a reasonable request.
